# Enhancing the Intrinsic and Extrinsic Stability of Halide Perovskite Nanocrystals for Efficient and Durable Optoelectronics

**DOI:** 10.1021/acsami.2c01822

**Published:** 2022-04-26

**Authors:** Clara Otero-Martínez, Nadesh Fiuza-Maneiro, Lakshminarayana Polavarapu

**Affiliations:** † Materials Chemistry and Physics Group, Department of Physical Chemistry Campus Universitario As Lagoas, CINBIO, Universidade de Vigo, Marcosende 36310, Vigo, Spain

**Keywords:** core−shell perovskite nanocrystals, encapsulation, MOF coating, metal oxide coating, polymer coating

## Abstract

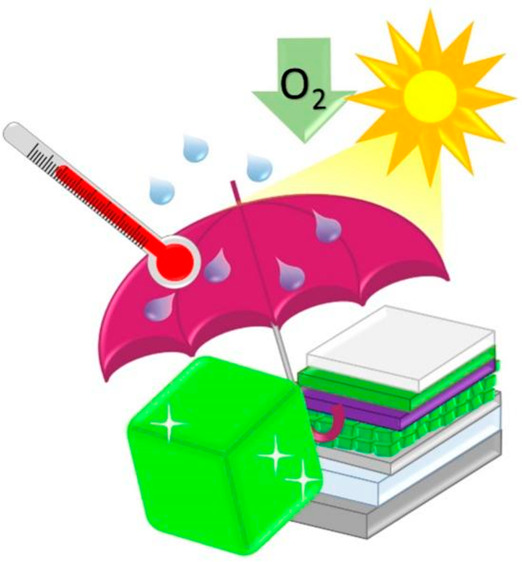

Over the past few years, metal halide perovskite nanocrystals have been at the forefront of colloidal semiconductor nanomaterial research because of their fascinating properties and potential applications. However, their intrinsic phase instability and chemical degradation under external exposures (high temperature, water, oxygen, and light) are currently limiting the real-world applications of perovskite optoelectronics. To overcome these stability issues, researchers have reported various strategies such as doping and encapsulation. The doping improves the optical and photoactive phase stability, whereas the encapsulation protects the perovskite NCs from external exposures. This perspective discusses the rationale of various strategies to enhance the stability of perovskite NCs and suggests possible future directions for the fabrication of optoelectronic devices with long-term stability while maintaining high efficiency.

## Introduction

Over the past decade, scientists from different disciplines, such as chemists, physicists, and engineers, have been amazed by the many exciting properties and potential applications of fascinating metal halide perovskites (MHPs).^1−3^ In addition to their intriguing properties (defect tolerance, long charge carrier diffusion lengths (>micrometers), high mobility compared to organic semiconductors, and high photoluminescence quantum yield), low cost, easy fabrication, and solution processability make them ideal candidates for optical and optoelectronic applications.^1^ Metal halide perovskites (MHPs) posses a formula of ABX_3_, where A is an organic or inorganic cation (methylammonium (MA^+^) and formamidinium (FA^+^) or Cs^+^) that sits in between octahedra made of divalent cations (Pb^2+^, Sn^2+^, or Bi^2+^) surrounded by six halide ions (X= Cl^–^, Br^–^, or I^–^). Although MHPs have been known since the late 1800s,^4^ they came into the spotlight in 2009 by the work of Kojima et al.^5^ who demonstrated the use of methylammonium lead halide as a photosensitizer in photoelectrochemical cells. The solid-state solar cell reported in 2012 with a power conversion efficiency (PCE) of more than 10% triggered the field of perovskite photovoltaics.^2^ These early reports drew the attention of researchers who were working on dye-sensitized solar cells, quantum dot solar cells, and organic solar cells. Since then, intense research has been carried out across the globe toward increasing the PCE, stability, and reproducibility of perovskite solar cells.^6^ This has led to a monotonic rise in the PCE of a single-junction solar cell from 10% to more than 25% in a short development time and is continuing to approach the theoretical efficiency limit (∼30%).^7^ The PCE of perovskite solar cells (centimeter scale) has already reached close to that of single-junction solar cells.^7^

On the other hand, colloidal metal halide perovskite nanocrystals (MHP NCs) have also been receiving increasing interest from the scientific community in parallel to thin-film perovskites.^1,8,9^ The high photoluminescence quantum yields (∼100%) and easy tunability of emission color by halide exchange make them excellent light sources for light-emitting applications.^1,3,10^ Unlike classical colloidal quantum dots (QDs), halide perovskite NCs does not require a high bandgap shell to passivate surface defects.^1,3^ The defect-tolerant nature of Br- and I-based perovskite NCs enabled obtaining them with near-unity PLQY at relatively low temperatures and using technical grade precursors.^1,3^ The colloidal synthesis of highly luminescent cesium lead halide perovskite (LHP) NCs reported by Protesescu et al.^11^ in 2015 and a few other early reports drew the attention of colloidal chemists, material scientists, spectroscopists, and device engineers working on classical-quantum dots (QDs).^1,12,13^ Since then, these classes of compounds have been virtually exploded regarding their synthesis, properties, and potential applications.^1^ Over the last 7 years, we have seen tremendous success and great progress in the field of halide perovskite NCs.^1^ The colloidal chemistry of perovskite NCs has been greatly advanced with improved understanding, and a wide range of facile synthesis methods have been developed for their shape and composition control.^1^ The optical properties of MHP NCs are controllable not only by their size but also through the composition of A, B, and X of ABX_3_.^1,14,15^ Especially, LHP NCs has become a leading candidate for next-generation light-emitting diodes and display technologies because of their high brightness, high color purity, tunable emission, high defect tolerance (green and red colors), and processability.^1^ The external quantum efficiency of LHP NC-based perovskite LEDs has surpassed more than 23 and 20% for green and red colors, respectively.^10,16^ In addition, it has been shown that they are promising for lasers, photodetectors, X-ray scintillators (they convert ionizing radiation into visible photons), phototransistors, and photocatalysis.^1^

Despite great progress in thin-film and NC-based LHP optoelectronics regarding the efficiencies, what stops their commercialization is the poor durability (besides efficiency of larger area devices and toxicity).^17−19^ But there are still many open questions that need to be answered regarding the lifetime of the optoelectronic devices made of this new class of materials. For example, silicon solar panels are expected to work for 25 years. However, the usage of perovskite optoelectronic devices for such a long runtime under harsh environmental conditions such as wind, rain, intense sunshine, and cold temperatures is being highly debated.^20^ Because of their low formation energy, perovskites are easy to make as well as easy to break (or degrade). In addition, the low crystal lattice energy leads to low formation energies of Pb and halide vacancies that destabilize perovskites via ion migration during device operation.^21^ This issue has been extensively summarized for thin-film perovskites in many review articles.^21,22^ In fact, both the thin-film and colloidal LHP NCs exhibit similar instability issues.^19,23^ Nevertheless, colloidal NCs have additional instability issues arising from the weak binding of ionic ligands with the NC surface.^3^ Therefore, the discussion in this perspective is mainly limited to colloidal halide perovskite NCs. One of the major challenges associated with halide perovskites to bring them from laboratory curiosity to real-world working devices is the enhancement of their intrinsic and extrinsic stabilities.

**Figure 1:**
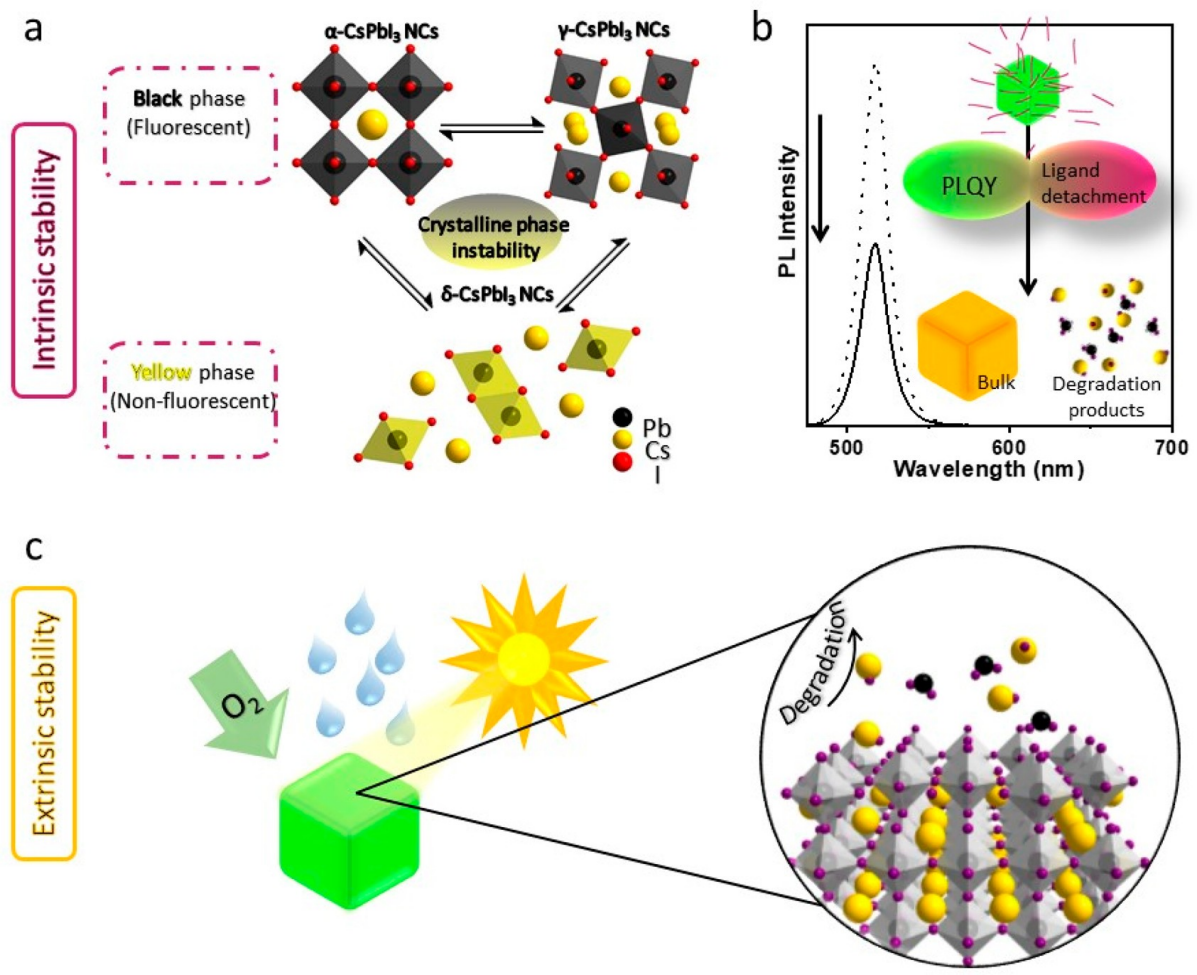
Intrinsic instability: (a) Schematic illustration of the transformation of black phase α (or γ)-CsPbI_3_ into yellow phase δ-CsPbI_3_ under ambient conditions. (b) Schematic illustration of the decrease in photoluminescence efficiency caused by the formation of defects through the detachment of ligands. This process can lead to the aggregation or degradation NCs. Extrinsic instability: (c) Schematic illustration of the degradation of MHP NCs under external stress such as oxygen, water (polar solvents), heat, and light. The soft ionic nature of perovskites causes their degradation when they encounter polar solvents. In addition, light illumination leads to ion migration in perovskites that causes irreversible degradation. Furthermore, the external factors often trigger the intrinsic instability of perovskites and thus accelerates the degradation process.

The intrinsic (or inherent) instability of LHP NCs is mainly of two types (Figure 1a, b): The first one is the transformation of the photoactive phase into the nonactive phase because of strain in the perovskite crystal lattice.^18,19^ This transformation is faster in the presence of external factors such as humidity. For instance, the black phase of α (or γ)-CsPbI_3_, which is photoactive, often transforms into a nonfluorescent yellow phase δ-CsPbI_3_ under ambient conditions.^18,19^ This transformation process is much faster in NC films (within a day) compared to the NCs in solution (generally, a few days to a month depending on the type of ligands). The second type of intrinsic instability of LHP NCs caused by the detachment of weakly bound surface ligands.^1,3^ The ligands often detach from NC’s surface with aging or by washing with polar antisolvents.^1,24^ This process can lead to aggregation or degradation of NCs. On the other hand, extrinsic instability refers to the instability of LHP NCs caused by external stress such as heat, oxygen, water (or polar solvents), or light (Figure 1c).^17,25^ It is worth mentioning that these external factors significantly affect the intrinsic stability of perovskites. For example, the phase transition of iodide perovskites is often accelerated by moisture and temperature. These factors can still influence the encapsulated devices because of the residual oxygen and moisture. Probably, encapsulation of devices under an inert atmosphere could help in this regard. The external effects can vary depending on the halide type and A-cation type. For example, it is well-known that Br-based LHP NCs exhibit better stability over iodide ones. Similarly, inorganic LHP NCs exhibit higher thermal stability compared to hybrid NCs.^26^ In fact, the external instability is also caused by the inherent soft and ionic nature of LHP NCs. Intense works have been carried out to understand the mechanism of environmental instability of LHP thin films;^17,25^ however, less investigated on colloidal NCs. It is most likely that the mechanism of degradation is similar in both cases.

The capping molecules (surface ligands) play a critical role in the stabilization and destabilization of NCs.^1,3^ It has been found that the ligands detach from the NC surface upon light illumination and thus the NCs aggregate into larger NCs.^26^ The light illumination initially leads to an enhancement in the PL intensity of perovskites, but it significantly quenches upon prolonged illumination due to defect formation, degradation, and morphological changes.^27^ The wavelength (or energy) of the light also has an influence on the degradation process. For example, UV light illumination effectively removes the surface ligands compared to visible light. It has been found that thinner LHP NCs such as quantum-confined nanoplatelets transform into nanowires or bulk NCs.^26^ In addition, light-induced negative effects can be worsened in the presence of oxygen, leading to photoinduced oxidation and then degradation of hybrid perovskites. A few studies have demonstrated the enhancement of the PL of LHP NCs upon short-time exposure to oxygen atmospheres.^28^ It was attributed to the deactivation traps created by photoexcitation. However, the photo-oxidation mechanism of inorganic LHP NCs over a long exposure time is still not clear.^29^ It is most likely that the reduction of PL of inorganic LHP NCs under a long exposure time to oxygen is mainly by the detachment of surface ligands and shape transformation.^29^ Considering the potentiality of LHP NCs in down conversion LEDs, in which blue light LEDs are used for generating other colors, it is very important to enhance the stability of NC films toward UV-light-induced morphological changes. On the other hand, the role of water (or humidity) in the degradation of LHPs is somewhat clear. The ionic nature of LHPs leads to their degradation upon contact with water. Although NCs are capped by ligands, their density and hydrophobicity are not enough to provide waterproofing to the surface of LHP NCs. In addition to the environmental factors, the thermal stability of LHPs is also one of the concerns. Interestingly, LHPs exhibit good thermal stability and it depends on the type of A-cation.^19^ Generally, inorganic LHP NCs exhibit higher thermal stability over hybrid ones. However, the decomposition induced by external factors such as oxygen and water can be accelerated and amplified at high temperatures. Therefore, the combination of heat and water can lead to the rapid degradation of LHP NCs. Therefore, all these instability issues need to be addressed for the fabrication of durable optoelectronic devices using LHP NCs.

## Strategies for Improving the Stability of LHP NCs

**Figure 2:**
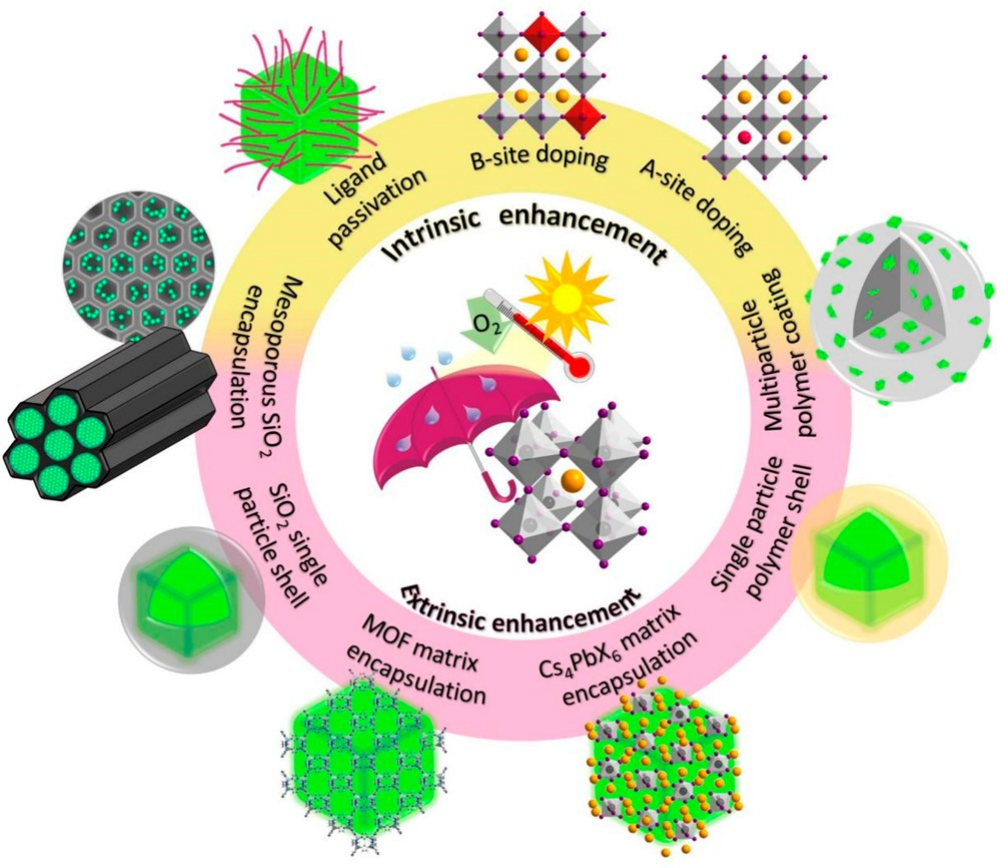
Schematic depiction of different strategies implemented for the intrinsic and extrinsic stability of halide perovskite NCs. Proper ligand passivation and doping improve the intrinsic stability, whereas the encapsulation with various shells such as metal oxide, polymer, metal–organic frameworks (MOF), Cs_4_PbX_6_ in the form of core–shell type architectures enhances the extrinsic stability. The encapsulation can lead to well-separated core–shell type NCs or multiple particles embedded shell matrix. It should be noted that the extrinsic enhancement often improves the intrinsic stability of perovskites.

To overcome the intrinsic and extrinsic instability of LHP NCs, researchers have developed various strategies over the years, and are illustrated in Figure 2. These strategies can be divided into three main categories: (1) Passivation with ligands that bind strongly to the NC surface, (2) doping (A or B-site), and (3) encapsulation (single particle or multiple particles). The surface passivation and doping mainly improve the structural phase stability,^18^ whereas the encapsulation shields the NCs from external stress such as heat, light, oxygen, and water (polar solvents).^1,30^ It should be noted that the protecting shells must be optically transparent to be used as light emitters in LEDs. The encapsulation strategies have been inspired by the methods previously used for the stabilization of colloidal metal NCs and classical QDs.^31^ Generally, colloidal NCs are often stabilized by making a robust shell structure on the NC surface in the form of core–shell NCs with controllable shell thickness. This has also been extended to LHP NCs by growing a shell of higher or lower bandgap with type I, II, or III band alignments.^30,32^ However, it is still challenging to achieve a uniform shell around an LHP NC surface without the agglomeration of cores or shells. This is because the shell structures often require the use of a polar solvent that is not compatible with LHP NCs. Despite a few challenges, core–shell type NCs have been successfully synthesized with greater stability in polar solvents in which pure LHP NCs disintegrate into precursors and other products. Another well-known strategy used for the stabilization and control of the dimensions of the LHP NCs is the in situ encapsulation in a mesoporous matrix, meaning that NCs are directly crystallized in a porous matrix with controllable pore size.^33,34^

## Stability Enhancement of LHP NCs by Ligand Engineering

**Figure 3:**
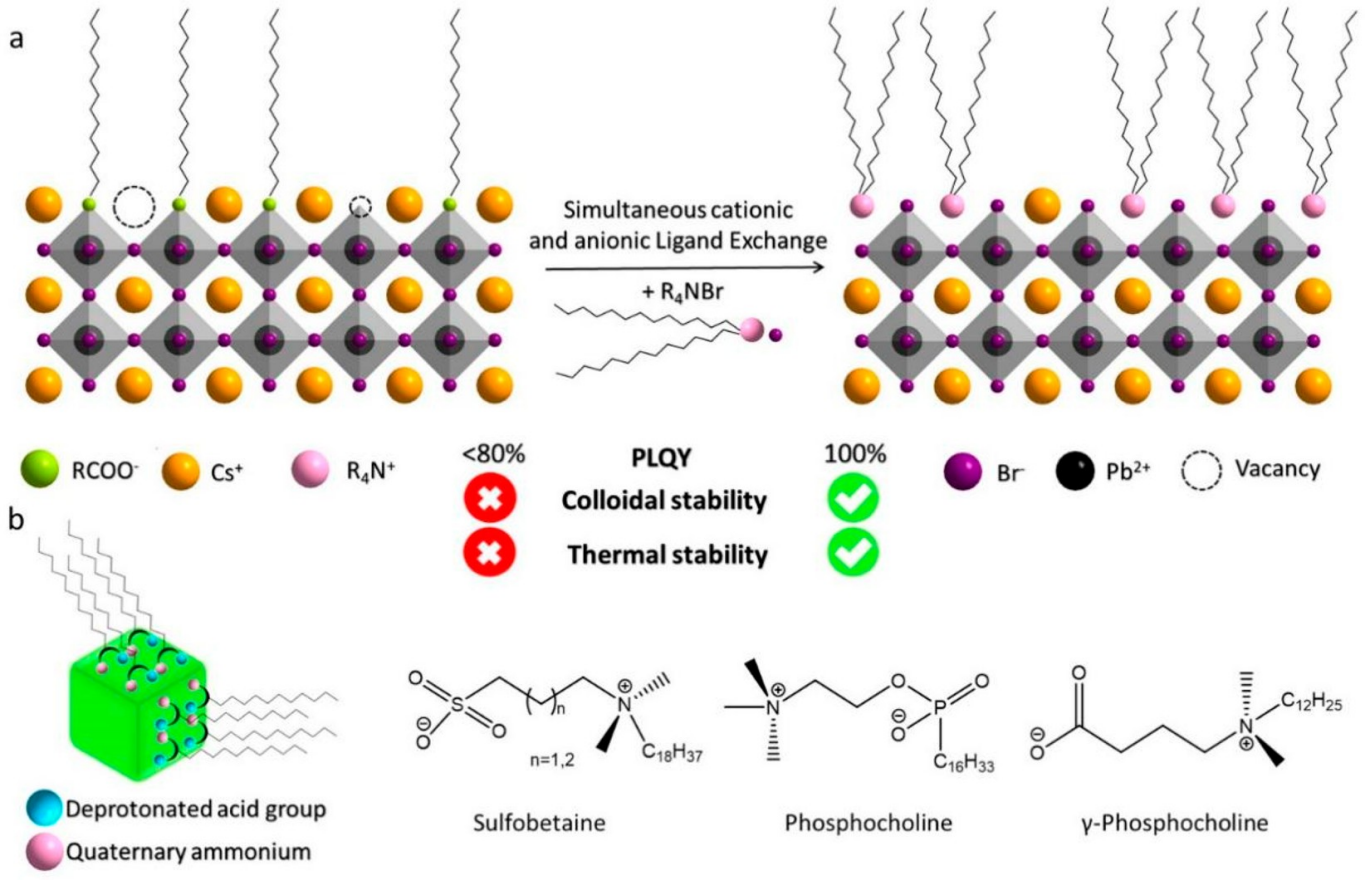
(a) Schematic illustration of the simultaneous cationic and anionic ligand exchange on CsPbBr_3_ NCs using quaternary ammonium ligands. Reproduced with permission from ref (38). Copyright 2019 American Chemical Society. (b) Schematic illustration of the zwitterionic molecule-capped LHP NCs and different kinds of zwitterionic molecules. Both the cationic and anionic parts of the ligands bind to the NC surface, providing a chelate effect for improved stability of the NCs. Reproduced with permission from ref (45). Copyright 2018 American Chemical Society.

The major factor that controls the colloidal stability of NCs is the surface chemistry of NCs and the strength of the interactions between NCs and ligands.^1,3^ The surface chemistry of LHP NCs is of special interest because of its relevance to the properties, making them vulnerable to instability and degradation, introducing new properties through surface functionalization. Most of the synthesis strategies for obtaining LHP NCs involve long-chain alkylamines and alkyl acids as ligands, with oleylamine and oleic acid (OAm/OA) as the most commonly used pair.^11^ The acid–base pair results in the formation of oleylammonium cations and oleate anions through proton transfer reaction. It has been widely accepted that the oleylammonium cations protect the NC surface by occupying some of the Cs atom positions, whereas the oleate neutralizes the surface charge but does not directly bind to the NC’s surface.^35^ Another hypothesis is that the oleate ions occupying the halide vacancies of the NC surface are more ionic in nature as compared to classical QDs. Therefore, ligand binding in LHP NCs is highly dynamic and maintains equilibrium with the excess ligands in the colloidal solution. Therefore, the ligands often desorb from the surface upon aging, dilution, or purification, thus leading to surface traps that result in a reduction of PLQY or even degradation.^3,35^ However, the PLQY and stability enhance upon adding an additional amount of OAm and OA to counter the ligand detachment.^1,36^ Another approach to overcome the instability caused by the transfer of the proton from the OA to the OAm is to carry out amine-free synthesis using OA and quaternary alkylammonium halides as capping ligands.^1,37^ The NCs prepared by this approach showed greater stability. For instance, Manna and co-workers demonstrated the simultaneous exchange of both cationic and anionic ligands on the surface of CsPbBr_3_ NCs using quaternary ammonium bromides (R_4_NBr) and the resultant NCs exhibit improved stability (colloidal and thermal) and high PLQY compared to CsPbBr_3_ NCs (Figure 3a).^38^ Similarly, Pan et al. proposed the formation of a protective enriched sulfide layer by employing didodecyldimethylammonium sulfide (DDA^+^S^2–^) as the surfactant, and the resultant NC films showed remarkable air stability.^39^ Nevertheless, recently several other types of ligands such as phosphonic acids,^40^ sodium dodecyl sulfate (SDS),^41^ dodecylbenzenesulfonic acid (DBSA),^42^ and dodecanethiol^43^ have been suggested for improving the colloidal stability of NCs. The strong interaction of alkyl phosphonic acids, thiolates, and thioethers toward Pb^2+^ ions leads to high affinity to the surface and to the passivation of trap states. For example, Wu et al.^44^ reported the synthesis of phase-stable CsPbI_3_ NCs trioctylphosphine–PbI_2_ (TOP–PbI_2_) as the reactive precursor and the resultant TOP-capped CsPbI_3_ NCs exhibit better stability compared to typical OLA/OA-capped CsPbI_3_ NCs. Similarly, the LHP NCs synthesized trioctylphosphine oxide also showed improved stability in polar solvents, as demonstrated by Zhang and co-workers.^44^

Some ligands of special interest are bidentate ligands such as succinic acid,^46^ 2,2′-iminodibenzoic acid,^47^*N*-methyl-2-pyrrolidon (NMP),^48^ and zwitterionic ligands^45,49^ which are stabilized by the chelate effect and show high efficiency in surface passivation and tight binding. For instance, Kovalenko and co-workers proposed the use of zwitterionic capping ligands for improved stability and durability of LHP NCs (Figure 3b).^45^ These ligands bind strongly to the LHP NC surface through the chelating effect, meaning that both the cationic and anionic parts of the ligand bind to the NC surface and thus provide greater colloidal stability (Figure 3b). Furthermore, bidentate ligands that form intermolecular interactions with one another can even protect the perovskites from water. For example, Nag and co-workers have shown that aromatic diamine ligands such as 4,4′-trimethylenedipyridine protects perovskites from water through the formation of long-range cation-π stacking.^50^ On the other hand, silane ligands such as 3-aminopropyl triethoxysilane (APTES)^51^ significantly improve the environmental stability, long-term storage, UV exposure, and resistance to polar solvents because of the formation of a cross-linking matrix by hydrolysis of the silyl ether groups, protecting the perovskite from the humidity. In conclusion, a huge variety of studies were reported, employing different capping ligands for the synthesis of perovskite NCs or as postsynthetic surface passivation agents, improving the stability and efficient light-emitting properties by passivation of surface defects.^1,3^ Despite significant progress in the synthesis of LHP nanocubes with improved stability, quantum-confined nanoplatelets (NPLs) still suffer from their poor stability and often tend to transform into thicker (bulklike) nanostructures within days after synthesis. In addition, most of the reported systems have been focused on Pb-based NCs, more works need to be devoted to the development of stable Pb-free perovskite NCs. It is important to note that although the ligands improve the intrinsic stability of perovskite NCs, their density and hydrophobicity are not enough to protect them from polar solvents, UV light, oxygen, and heating.

## Stability Enhancement by A and B-Site Doping/Alloying

**Figure 4:**
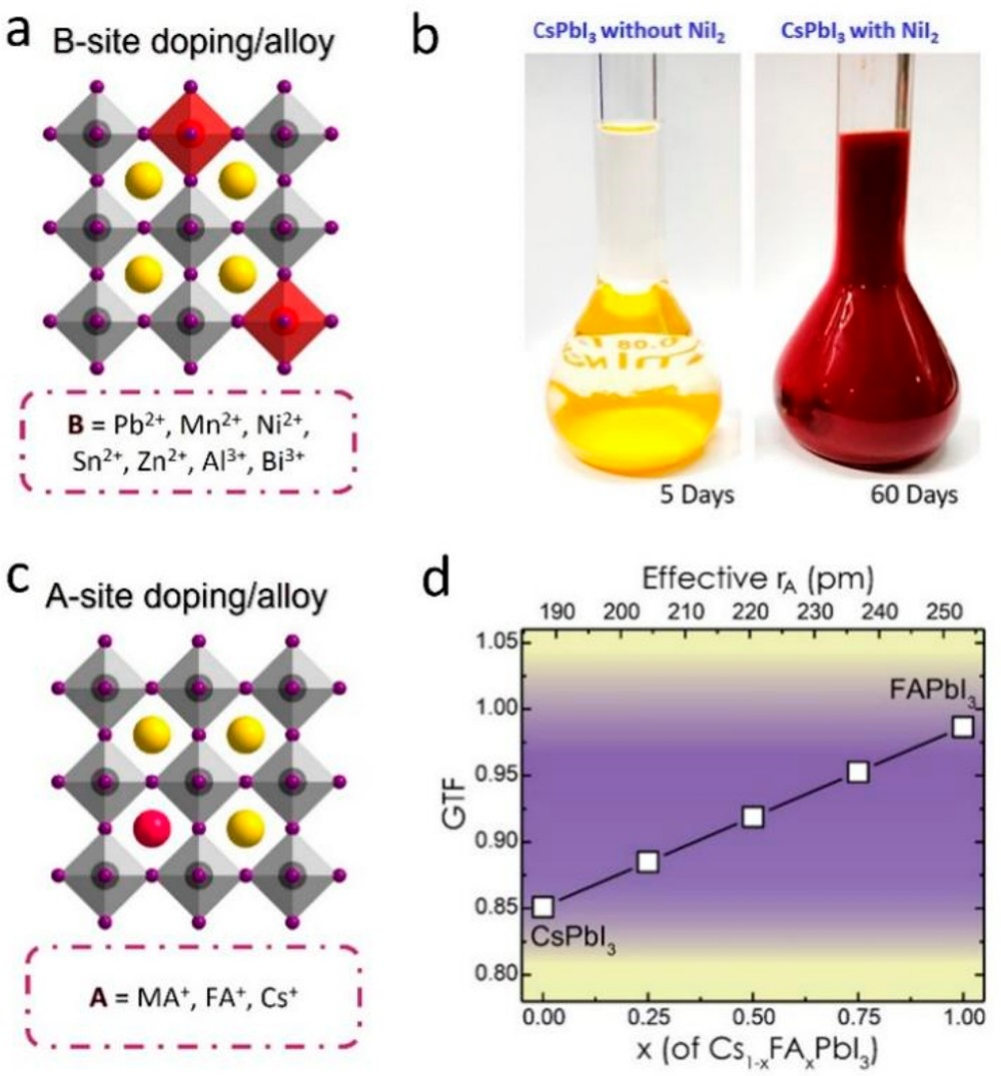
(a) Schematic illustration of mixed B-cation typical cubic crystal structure. (b) Photograph of colloidal solutions of undoped and Ni(II) doped CsPbI_3_ NCs after 5 and 60 days, respectively. Reproduced with permission from ref (54). Copyright 2019 American Chemical Society. (c) Schematic illustration of mixed A-cation cubic crystal structure. (d) Goldschmidt tolerance factor (GTF) vs concentration of FA^+^ ion shows that all compositions of Cs_1–*x*_FA_*x*_PbI_3_ (*x* = 0–1) are expected to be phase-stable. The top axis shows the effective A-site radius. Reproduced with permission from ref (14). Copyright 2018 American Chemical Society.

Doping (or alloying) in LHP NCs has been heavily investigated not only to induce new optical features but also to improve colloidal stability and PL efficiency (Figure 4).^1,3^ In principle, in the case of doping, the amount of dopant should be very low (at least less than 1%), otherwise, it should be called an alloy. However, in many perovskite NC papers, it is often called doping regardless of the amount of dopant.^1^ Although the doping/alloying strategy has been implemented to enhance the stability of perovskite NCs of all three halides (X = Cl, Br, and I), here we focus mainly on the iodide system because of its relevance to photovoltaics and red-emitting LEDs.^1,19^ Importantly, iodide-based perovskite systems are the ones that exhibit the least phase stability and it is one of the most challenging problems to be solved.^19^ Despite their great potential in optoelectronic applications, FAPbI_3,_ and CsPbI_3_ perovskites tend crystallize into the photoinactive yellow (δ-) phase at room temperature because it has the lowest free energy of formation energy.^52,53^ Therefore, the photoactive black phase of iodide perovskite NCs often transforms into an optically inactive yellow phase upon aging the colloidal solution for a few days or exposure to moisture. For instance, the as-synthesized CsPbI_3_ NCs generally exhibit high PLQY; however, they become nonfluorescent after a few days of preparation.^54^

One of the main reasons for this phase transform is its relatively low tolerance factor (<0.8), which can be compensated by the lattice contraction through doping with small size B cations.^9^ In theory, substituting Pb^2+^ ions with a smaller B-cation or replacing Cs with a bigger cation can stabilize the black phase α-CsPbI_3_ by reducing the [BX_6_]^4–^ octahedral tilt. For more details on the relation between the Goldschmidt’s tolerance factor, B-site doping, and phase stability, we refer the readers to the previous review articles.^19,55^ Over the years, many studies have been focused on improving the phase and thermal stability of CsPbI_3_ NCs by A- and B-site doping/alloying with various monovalent (MA^**+**^, FA^**+**^, Rb^**+**^, Na^**+**^, K^**+**^) and divalent metal cations (Mn^2+^, Ni^2+^, Sn^2+^, Zn^2+^, and Sr^2+^), respectively (Figure 4a, c).^1,54,56,57^

In addition, trivalent cation (Al^3+^, Bi^3+^, and lanthanide) doping has been applied to improve the phase stability of iodide perovskites.^58,59^ However, it is still not clear whether the trivalent cations really incorporate into the perovskite lattice. Generally, doping/alloying in perovskite NCs can be achieved by direct synthesis as well as a postsynthetic treatment with corresponding metal precursors. To the best of our knowledge, postsynthetic doping/alloying offers better control over the dopant concentration in the lattice. For instance, Zou et al.^60^ demonstrated the stabilization of CsPbX_3_ crystal lattice by doping/alloying with Mn^2+^ cations through postsynthetic treatment with MnX_2_ precursor. The prepared NCs with an optimum dopant concentration showed enhanced thermal and phase stability under ambient conditions. Generally, OLA/OA-capped CsPbI_3_ NCs exhibit poor stability and often turn into a non-fluorescent yellow phase within a few days after synthesis (the stability may vary from batch to batch); however, they can be stable for more than 2 months after doping its lattice with smaller cations like Ni^2+^ (Figure 4b).^54^ The enhanced stability was attributed to the lattice contraction caused by the shortening of the metal-I bond.^54,61,62^ Interestingly, in most cases, the bandgap of CsPbI_3_ remains unaltered regardless of the dopant.^54,61,62^ However, alloying with Sn^2+^ can alter its bandgap, and the bandgap of CsPb_*x*_Sn_1–*x*_I_3_ alloy NCs gradually decreases with increasing the amount of Sn^2+^ content.^57^ Furthermore, the doping/alloying strategy has been extended to Br- and Cl-based perovskites to improve their optical properties.^3,63,64^ In general, Cl-based perovskites exhibit very low PLQY because of their defect-intolerant nature. Nonetheless, few reports demonstrated the significant improvement in the PLQY of CsPbCl_3_ NCs by doping with other metal cations or halide passivation.^3,63^ However, it is still unclear whether the enhancement of PLQY is due to filling of halide vacancies or metal ion doping. On the other hand, A-site alloying has been relatively less explored for phase stability enhancement of perovskite NCs.^14,56,65^ The increase in the tolerance factor of CsPbI_3_ NCs has been inspired from the studies of mixed A-cation perovskite thin films that exhibit enhanced phase stability compared to that of monocation perovskite films (Figure 4c).^14^ In contrast to B-site doping/alloying, mixed A-cation perovskite NCs have been mostly obtained through postsynthetic A-cation cross-exchange. In fact, the A-site cation exchange is energetically more favorable compared to B-site cation exchange. By mixing colloidal NC solutions of CsPbX_3_ and FAPbX_3_ in different ratios, one can finetune the A-site cation composition and thus the tolerance factor to obtain a stable perovskite phase (Figure 4d).^14^ The increase in the A-cation size reduces the PbI_6_^4–^ octahedra tilting, maintaining the photoactive cubic phase. Recently, these mixed A-cation perovskite NCs have received significant interest as photosensitizers for the fabrication of solar cells with long-term stability.^14,56,65^ However, it is challenging to characterize the composition of mixed-cation perovskite NCs. In most previous reports, the stability of iodide-based LHP NCs has been studied by alloying either A-cation or and B-cation. Future studies could be focused on simultaneously alloying of both A and B-cations. In addition, X-site doping/alloying can significantly improve the stability of LHPs. For example, doping of iodide perovskites with a small amount of Br or Cl can significantly improve their stability. In fact, Br and I mixed perovskites along with mixed A-cations have been extensively implemented in the fabrication of relatively stable perovskite solar cells.^66^ Recently, this concept has been extended to perovskite NC-based solar cells to obtain relatively stable solar cells.^56^

## Encapsulation of Perovskite NCs

**Figure 5:**
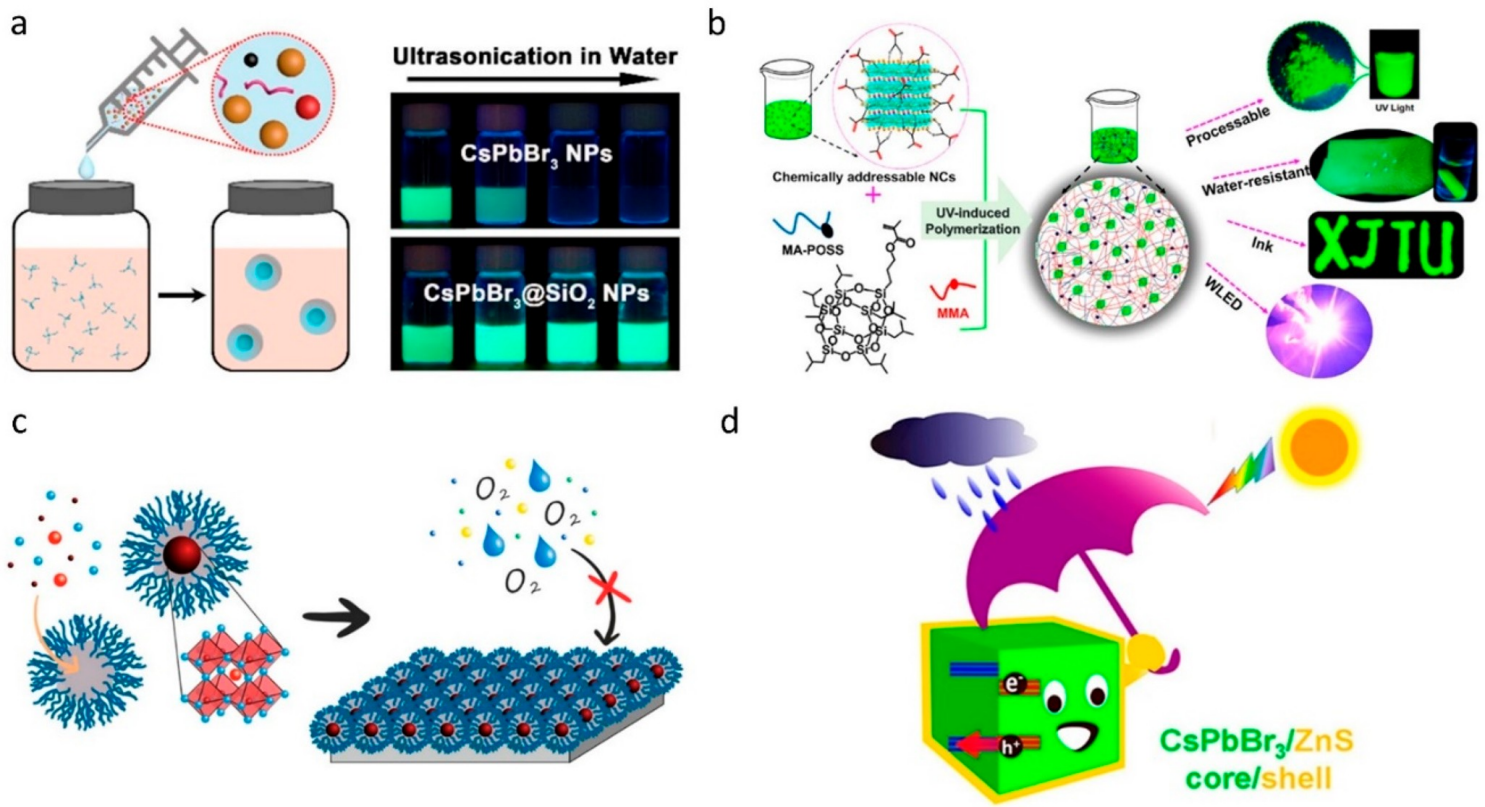
(a) (Left) Schematic illustration of the synthesis of CsPbBr_3_ @SiO_2_ core–shell NCs, where the precursors dissolved in DMF are injected into a nonpolar organic solvent in the presence of tetramethoxysilane (TMOS). (Right) Photographs of the colloidal solutions of pristine CsPbBr_3_ NCs and CsPbBr_3_@SiO_2_ core–shell NCs under UV light illumination. Reproduced with permission from ref (67). Copyright 2018 American Chemical Society. (b) Encapsulation of surface-functionalized CsPbBr_3_ NCs in a polymer matrix through UV-light-induced polymerization. The NCs and the corresponding films are compatible with water. Reproduced with permission from ref (68). Copyright 2018 American Chemical Society. (c) Schematic illustration of the synthesis of block copolymer encapsulated LHP NCs for enhanced stability against water and oxygen. Reproduced with permission from ref (69) Copyright 2019 American Chemical Society. (d) Schematic illustration of CsPbBr_3_/ZnS core/shell NC that are stable against water and light. Reproduced with permission from ref (70). Copyright 2020 American Chemical Society.

Although the intrinsic stability of LHP NCs can be greatly improved by ligand engineering and doping/alloying, they are still prone to degradation in exposure to water, intense light illumination, oxygen, and heating. To overcome this problem, the surface of the NCs needs to be protected with materials that chemically and physically prevent the water and oxygen from reaching the NC surface. A wide range of materials including metal oxides, polymers, MOFs, metal chalcogenides, and perovskite derivatives have been used as shells to protect the surface of perovskite NCs (see Figure 2).^1,30^ The encapsulated perovskite NCs are of two types: (1) single core–shell colloidal NCs, (2) multiple NCs incorporated into a shell matrix. Among various encapsulants, SiO_2_ has received significant interest because of its chemical and thermal stability along with low toxicity. The SiO_2_ shell has been extensively applied to metal NPs and classical semiconductor QDs to stabilize the polar solvents. However, coating SiO_2_ shells on LHP NCs is challenging because the hydrolysis reaction requires some amount of water that can damage the NCs. Several attempts have been made to coat the SiO_2_ on LHP NCs using a minimum amount of water or no water, yielding multiple NC-embedded SiO_2_ matrices.^71^ Another approach that is often used is the postsynthetic encapsulation of multiple NCs in a preprepared mesoporous silica matrix by incubating them together for a few hours. The studies showed that mesoporous encapsulation enhances the stability of LHP NCs and prevents the halide ion exchange when NCs of two different halides are mixed, enabling the fabrication of white LEDs.^72^ Besides, the NCs can be directly grown in the pores of mesoporous fibers by introducing corresponding precursors into the porous followed by heating or the addition of an antisolvent.^33,34^ However, it is still not clear whether the mesoporous structure provides full waterproofing because the water molecules can go into the pores to destroy the NCs. A few reports demonstrated the fabrication of LHP@SiO_2_ core–shell NCs by reprecipitation of LHP NCs in the presence of tetramethoxysilane (TMOS), which is a precursor for silica coating (Figure 5a).^67^ The NCs completely covered with SiO_2_ shells clearly exhibit water stability.^73^ The SiO_2_ shell at the single-particle level has also been achieved by interfacial synthesis, in which the coating takes place at the water-hexane interface by mixing TMOS and Cs_4_PbX_6_ NCs. This reaction leads to the stripping of CsX and SiO_2_ coating simultaneously and results in the formation of CsPbX_3_@SiO_2_ Janus or core–shell NCs.^74^ The strategies reported for encapsulation of LHP NCs in the SiO_2_ matrix have been extended to other oxides such as TiO_2_, Al_2_O_3_, and ZnO as well as metal–organic frameworks (MOFs).^74−77^ In addition, polymer materials have been used for the efficient encapsulation of LHP NCs. The LHP NC–polymer composites can be prepared either by mixing NCs with polymers or through polymerization on the NC surface. For instance, Pan et al. prepared CsPbBr_3_–polymer inks by initiating the polymerization on the NC surface through their surface functionalization, and the composite NCs exhibit water stability (Figure 5b).^68^ On the other hand, LHP–polymer core–shell NCs have been obtained either through in situ synthesis in hydrophobic pores of block copolymers (Figure 5c)^69^ or by phase transfer using polyzwitterionic ligands.^49^ In addition, perovskite NC@polymer core–shell structures can be obtained through the polymerization of photoactive monomeric ligands on the NC surface.^78^ In all these cases, the core–shell NCs exhibit stability against water (moisture), oxygen, and heat. The shells not only stabilize the perovskite NCs against external factors but also prevent the halide cross-exchange between NCs. For example, Ravi et al. demonstrated the suppression of halide exchange in LHP NCs though PbSO_4_–oleate capping. Despite improved stability, the shells block the charge transport between NCs and thus limit their applications.^79^ Recently, there has been growing interest in coating LHP NCs with metal chalcogenides to enhance the stability as well as to induce new functions (Figure 5d).^70^ Despite few successful demonstrations, it is still challenging to obtain such core–shell NCs because of lattice mismatch between LHP and metal chalcogenides.

## Durable Optoelectronic Devices through Encapsulation

**Figure 6:**
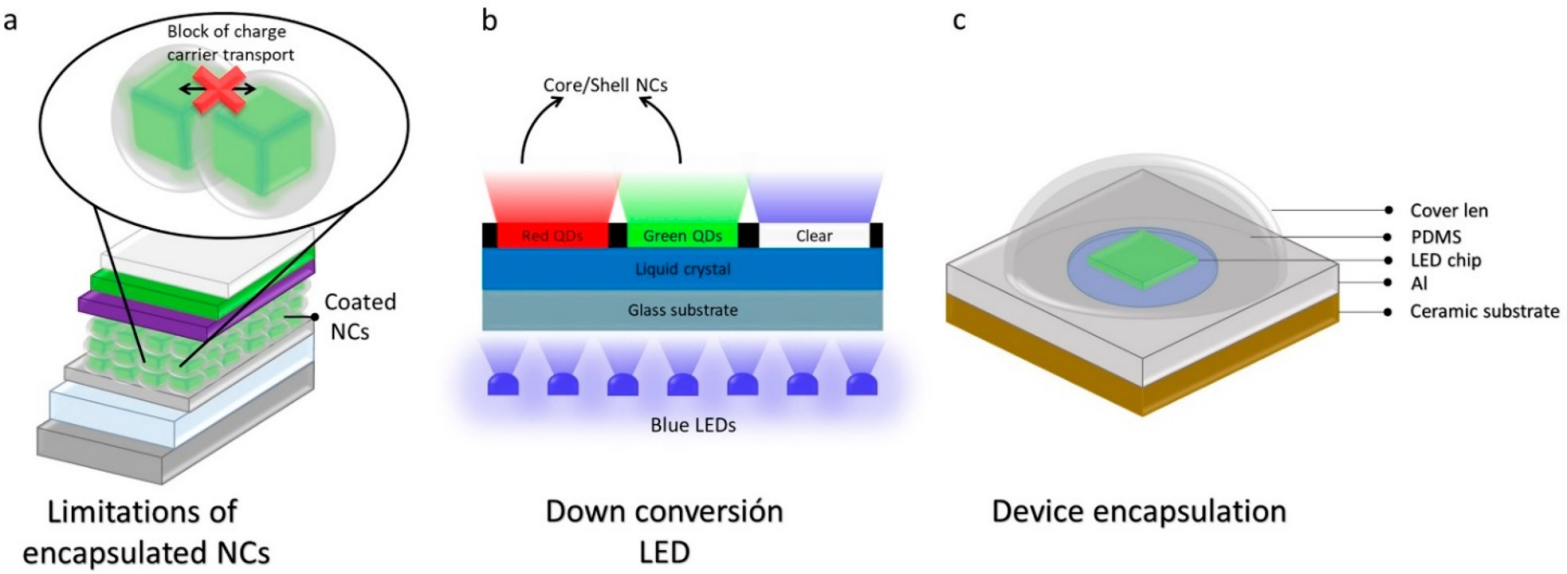
(a) Schematic illustration of an optoelectronic device in which encapsulated NCs are integrated as active material. The dielectric shell around the NCs blocks the charge transport between neighboring NCs and thus limits their use in solar cells and electroluminescent devices. (b) Schematic illustration of color conversion device based on encapsulated LHP NCs, where blue LEDs is used as backlight to excite the NCs of different colors. (c) Schematic illustration of an encapsulated optoelectronic device made using ligand capped LHP NCs as the active medium. Panel C is reproduced with permission from ref (93). Copyright 2019 Springer Nature.

The ultimate goal of the intrinsic and extrinsic stability enhancement of LHP NCs is to use them as active semiconductor materials in the fabrication of durable optoelectronic devices with long-term stability. The encapsulation of LHP NCs in inert shells such as SiO_2_ limits their use in optoelectronic devices. For instance, they cannot be use as photosensitizers in solar cells or charge recombination medium in electroluminescent devices because the inert shells around the NC surface block the change transport between adjacent NCs (Figure 6a). However, they are potential light sources for down conversion LEDs, LED backlit NC color conversion, NC color enhancement films, and NC color converters for high-definition display applications, in which the semiconductor NCs are illuminated with UV light (Figure 6b).^72^ In addition, the ligand stabilized colloidal NCs with improved intrinsic stability could be used in solar cells and electroluminescent devices;^1^ however, the devices need to be encapsulated with proper materials to protect them from oxygen, water, intense light, and heat-induced degradation (Figure 6c).,^80^ A proper encapsulation of the sensitive photoactive layers of solar cells is reflected in a considerable increase in the device lifetime.^81^

The encapsulants need to have good insulation properties, possess high light transmittance, and prevent the ingress of moisture and oxygen into the device.^82^ In addition, the encapsulation technique must be easy to perform and cost-effective.^83^ In this regards, glass offers excellent protection and a high optical transmittance to the entire UV spectrum.^84^ However, encapsulation with rigid glass is not suitable for flexible devices and roll-to-roll encapsulation processes. On the other hand, flexible polymer encapsulation of devices consists of barrier material on the top and the bottom bonded with an adhesive.^85^ The adhesives used in flexible polymer encapsulations can be sensitive to temperature^86^ and UV light incidence.^87^ Several investigations were carried out to determine the stability by sealing with adhesives.^88,89^ Furthermore, thin-film encapsulation is a promising technique to enhance the long-term stability of devices and it has the advantage of direct deposition on flexible devices without the use of barrier adhesive materials.^90^ The thin-film encapsulation can be single or multilayer, and a wide range of organic and inorganic materials can be used. It has been shown that in many cases a single layer is insufficient for effective encapsulation because it is difficult to avoid the formation of cracking and pinholes on the surface of the layer. High efficacy has been reported by encapsulating in alternating inorganic and organic multilayer films because of the combined effects of both materials.^85^ The inorganic layers improve the stability of the device by increasing the blockage of moisture and oxygen, whereas the organic layers can be used to improve the long-term stability of blocking layers.^91^ Nevertheless, the insertion of buffer layers between the transfer and active layers enhances the stability and reduces residual stress and interfacial defects.^92^ Several techniques including sputtering, atomic layer deposited (ALD), and chemical vapor deposition (CVD) have been developed for the deposition of thin films that provide protection against harsh environmental factors.^94,95^

Considering the sensitivity of the devices to temperature, and the high temperatures used in the deposition of thin films at low temperatures, it has been essential to develop new strategies for thin-film deposition at low temperatures.^96^ Additionally, ALD methods are characterized by the need to be carried out in high-vacuum conditions. Consequently, few open-air studies were reported, employing silica layers, plasma-deposited multilayer thin-film barrier,^97^ or perhydropolysilazane (PHPS) ink.^98^ Another potential encapsulant is graphene, which is a good barrier material because of its permeation properties.^99^ All these techniques and encapsulants have been heavily investigated to improve the durability of perovskite solar cells; however, in principle, these are also applicable to NC-based optoelectronic devices.

## Summary and Outlook

Metal halide perovskite NCs have been emerged as leading candidates for optoelectronic applications because of their interesting optical and optoelectronic properties. Especially, the high PLQY, high absorption coefficient, facile tunability of emission color by halide composition, and narrow line width make them potential light sources for LEDs and display applications. In addition, recently, there has been a growing interest in using them as photosensitizers in solar cells because of their higher stability compared to thin-film counterparts. Over the years, we witnessed a rapid growth in the field regarding the shape-controlled synthesis and the understanding of their photophysical properties and their application in optoelectronic devices. The efficiency of LHP NC-based LEDs and solar cells has been on the rise and rapidly approaching the theoretical efficiency. However, the intrinsic and extrinsic instability of LHP NCs limits the fabrication of durable optoelectronic devices. Here, intrinsic instability refers to the instability caused by crystal structure tolerance and the ligand–NC surface interactions. On the other hand, the extrinsic instability refers to the degradation of perovskite NCs on exposure to external factors such as water, heat, oxygen, and intense light illumination. Because of the strong ionic character, perovskites can degrade as easily as they form. It is important to note that the external factors greatly influence the intrinsic stability of perovskites, often accelerating the phase transition and degradation process.

Over the years, various strategies have been developed to improve both the intrinsic and extrinsic stability of LHP NCs. These strategies are ligand engineering, doping and encapsulation in a matrix. It has been found that the doping and strong NC-ligand binding improves the optical and phase stability of LHP NCs, while the encapsulation protects them from water, oxygen, light, and high temperature. Despite great progress in achieving perovskite NCs with improved stability, there are several outstanding challenges that remains unanswered: 1) Extension of the encapsulation strategies to Pb-free MHP NCs. Considering the poor stability of Sn-based perovskite NCs, more efforts need to be devoted toward the encapsulation of tin halide perovskite NCs for improving their intrinsic and extrinsic stability. 2) Encapsulation of quantum-confined nanocrystals such as nanoplatelets that are rather unstable compared to bulk-like nanocubes. 3) Currently, most of the synthesis methods yield significant percentage of composites made of multiple particles encapsulated shell matrix. Therefore, the surface coating strategies need to be improved to achieve core–shell particles with improved yield. Importantly, a systematic study is required to understand the improvement of stability against external stress for each encapsulating material.

Recently, there has been growing interest in the situ synthesis of encapsulated LHP NCs directly on desired substrates.^100^ Despite improving the stability, the dielectric shells on the surface of NCs limit their use in photoelectrochemical cells and electroluminescent devices because the dielectric shells block the charge transport between NCs. Future studies could be focused on the preparation of conjugated polymer-coated LHP NCs improving the transport properties in corresponding films. Nevertheless, the encapsulated LHP NCs looking promise down-conversion LEDs and ultrahigh definition display applications. On the other hand, ligand-capped NCs without encapsulation can be integrated into solar cells and electroluminescent cells; however, the devices need to be encapsulated with proper materials to enhance their durability. So far, most studies have been focused on improving the efficiency of small-area (centimeter-scale) devices using ligand-capped LHP NCs as active medium. Therefore, future studies could be focused on fabrication of large-area devices with long-term stability.
